# Metoclopramide Test in Hyperprolactinemic Women With Polycystic Ovarian Syndrome: Old Wine Into New Bottles?

**DOI:** 10.3389/fendo.2022.832361

**Published:** 2022-02-18

**Authors:** Claire Rodier, Blandine Courbière, Sara Fernandes, Marie Vermalle, Bretelle Florence, Noémie Resseguier, Thierry Brue, Thomas Cuny

**Affiliations:** ^1^ Department of Gynecology-Obstetric and Reproductive Medicine, AP-HM, Hôpital La Conception, Marseille, France; ^2^ CNRS, IRD, IMBE, Avignon Université, Aix Marseille Univ, Marseille, France; ^3^ Aix Marseille Univ, APHM, Hôpital de la Timone, Service d’Epidemiologie et d’Economie de la Santé, Unité de Recherche Clinique, Direction de la Recherche en Santé, Marseille, France; ^4^ EA3279, CEReSS, Health Service Research and Quality of Life Center, Aix-Marseille University, Marseille, France; ^5^ Aix Marseille Univ, APHM, Marseille Medical Genetics, Inserm U1251 and Hôpital de la Conception, Service d’Endocrinologie, Marseille, France

**Keywords:** PCOS, hyperprolactinemia, hyperandrogenism, oligo-anovulation, metoclopramide test

## Abstract

**Introduction:**

Polycystic ovarian syndrome (PCOS) is the most frequent etiology of anovulation, hyperandrogenism and infertility in women. Its pathophysiology remains poorly elucidated. Hyperprolactinemia (hPRL) is common in women of reproductive age and may partially mimic the clinical phenotype of PCOS. The simultaneous finding of both conditions is therefore not rare, however there are conflicting studies on whether a link exists between them.

**Materials and Methods:**

We conducted a retrospective monocentric study between 2015 and 2021 and among women who were referred for possible PCOS, we selected those who met the ESHRE/Rotterdam definition criteria. hPRL was defined as two values above the upper limit of normal with at least one measurement in our centre.

**Results:**

A total of 430 women were selected, of whom 179 met the PCOS criteria. 50 out of 179 patients (27.9%) had at least one elevated value of PRL and 21 (11.7%) had hPRL according to our definition. Among the 21 women of the PCOS/hPRL cohort, 5 (23.8%) had a microprolactinoma and all of them had PRL level ≥ 60 ng/ml. The remaining cases were macroprolactinemia (n=5), iatrogenic hPRL (n=4), primary hypothyroidism (n=1) or unexplained (n=6) despite exhaustive investigations. The metoclopramide test resulted in an increase of basal PRL < 300% in all prolactinomas and ≥ 300% in all the other etiologies.

**Conclusion:**

hPRL was a common finding in PCOS women, secondary to a microprolactinoma in a quarter of cases. Metoclopramide test performed in women with hPRL below 60 ng/ml appeared as a helpful tool 1) to discriminate pituitary causes from others etiologies, 2) to potentially avoid unnecessary pituitary MRI.

## Introduction

Polycystic ovarian syndrome (PCOS) is the most frequent etiology of anovulation, hyperandrogenism and infertility in women. Indeed, between 2.2 and 21.3% of women in reproducing age are diagnosed with this pathology (1), with up to 70% of affected women undiagnosed (2). Both oligo-anovulation and hyperandrogenism constitute the cardinal features of PCOS, while insulin resistance and excess weight largely contribute to its pathogenesis, clinical/biochemical manifestations, and long-term sequelae (3).

Despite this high rate and an extended time of study (PCOS has been described since 1935 when Irving Stein and Michael Leventhal identified an association between amenorrhea and bilateral polycystic ovaries), the pathophysiology of PCOS remains unclear. Hyperprolactinemia (hPRL), defined as an excess of prolactin in blood above laboratory reference limits, is also common in women of reproductive age and may mimic the clinical phenotype of PCOS. The prevalence of hPRL is hard to accurately assess, however it could concern up to 4% of women (4). When facing hPRL, pregnancy, breastfeeding, medications, and the presence of macroprolactinemia must be ruled out in priority. The latter results from the formation of prolactin-immunoglobulin complex, and was recognized as a frequent cause of hPRL since many years (5), with an incidence as high as 25% in some series (6). It is, however, usually asymptomatic due to the limited bioavailability and bioactivity of the macromolecule. Finally, pathological causes like hypothyroidism, renal insufficiency and pituitary adenomas (mainly prolactin-secreting phenotype, PRLomas) represent the remaining causes encountered in clinical practice. PRLomas account for as much as 60% of pituitary adenomas (7), microadenomas in young women being the most typical presentation. On the other hand, the simultaneous finding of a hPRL and PCOS is not rare, with a reported prevalence of hPRL ranging between 2 and 22% in PCOS women (8, 9). Therefore, distinguishing hPRL due to PRLomas from other causes of hPRL is relevant, as it could, in case of a PRLoma, result either in the prescription of dopamine agonist therapy, or even pituitary surgery (10). This is of upmost importance in case of infertility, where combined treatment for PCOS and hPRL are actively discussed, in order to restore menstrual cycles and a regular qualitative ovulation.

Therefore, the aim of this study was to determine the prevalence of hPRL among a large cohort of well-characterized PCOS women, and to further describe the causes of hPRL in this context.

## Material And Methods

### Study Design

**Figure 1:**
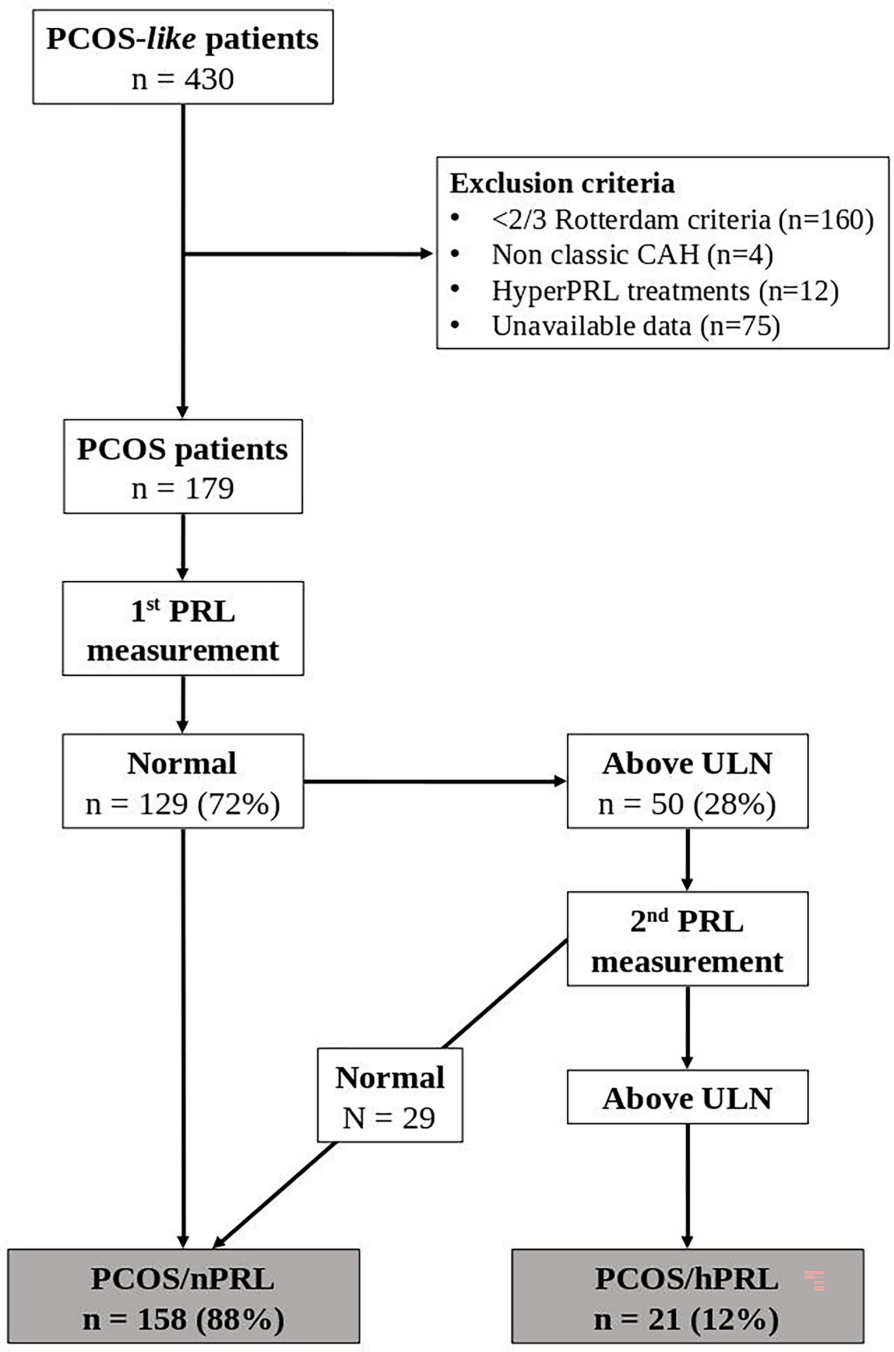
Flow Chart of the Study. CAH, Congenital Adrenal Hyperplasia; hPRL, hyperprolactinemia; nPRL, normoprolactinemia; PCOS, Polycystic Ovary Syndrome; PRL, Prolactin; ULN, Upper Limit of Normal.

We conducted a retrospective monocentric study of patients admitted between January 2015 and July 2021 at the University Hospital La Conception in Marseille, either in the day hospital of the Endocrinology Department or in the Medically Assisted Procreation Department. Women with clinical symptoms suggestive of PCOS (oligo-anovulation, hyperandrogenism, menstrual irregularities, hirsutism, acne) were selected in our computer database (**Figure 1**). We, then, selected only women who met the diagnosis criteria of PCOS used by the European Society of Human Reproduction and Embryology (ESHRE), established in 2018 (**Table 1**) (11), even for women diagnosed before 2018. According to these criteria, four different phenotypes of PCOS are described: A, when the 3 PCOS criteria are met, B, for ovulatory dysfunction and hyperandrogenism, C, for hyperandrogenism and polycystic ovarian morphology, and, D, for ovulatory dysfunction and polycystic ovarian morphology. Women were respectively classified as being underweight (BMI < 18.5 kg/m^2^), of normal weight (18.5 ≤ BMI < 25 kg/m^2^), overweight (25 ≤ BMI < 30 kg/m^2^) or obese (BMI ≥ 30 kg/m^2^).

**Table 1:** Diagnosis criteria of PCOS used by the European Society of Human Reproduction and Embryology (ESHRE), established in the Rotterdam consensus in 2018.

Criteria	Details
Irregular cycles and ovulatory dysfunction (OD)	Irregular menstrual cycles are defined as: • normal in the first-year post menarche • > 1 to < 3 years post menarche: < 21 or > 45 days • > 3 years post menarche: < 21 or > 35 days or < 8 cycles per year • > 1 year post menarche > 90 days for any one cycle
Biochemical or clinical hyperandrogenism (HA)	• Elevated levels of testosterone, Androstenedione or dehydroepiandrosterone sulfate (DHEAS) •Acne, alopecia, hirsutism
Polycystic ovarian morphology (PCOM)	At least 1 of the 2 ovaries presenting: •>= 20 follicles using endovaginal ultrasound transducers with a frequency bandwidth that includes 8MHz •Volume>10 mL •Surface >5,5cm2
At least 2 criteria out of the 3 **After exclusion of non-classic congenital adrenal hyperplasia, Cushing’s, adrenal or ovarian virilizing tumors.**	

All the women enrolled in this study underwent a standardized clinical, biochemical and imaging work-up at our centre. LH, FSH, prolactin (PRL), antimullerian hormone (AMH), 17-OH-progesterone, estradiol, testosterone, delta 4 androstenedione and dehydroepiandrosterone sulfate (DHEAS) were measured in the plasma. PRL chromatography was performed to rule out macroprolactinemia or big prolactin only if the basal PRL value was elevated. Women with a single high value of PRL were systematically candidate to a second measurement or asked for a previous PRL value (**Figure 1**). We considered patients as being hPRL, if they had at least two measurements of PRL, including one performed in our centre, above the upper limit of normal (ULN).

Women were excluded from the study in case of pregnancy, current breastfeeding, other causes of hyperandrogenism (Cushing’s syndrome, classic or non-classic congenital adrenal hyperplasia), current intake of hPRL drugs (including estrogen/progestin birth control pill) or interrupted less than 4 weeks before admission to the hospital. Accordingly, we paid a careful attention that none of our patients were taken medications or even supplements known to interfere with the PRL and/or the dopamine receptor system. Systemic disease known to increase PRL (inflammatory diseases, renal insufficiency, or hypothyroidism) were also ruled out. For hypothyroidism, all the women had contemporary measurement of TSH (normal range: 0.27 - 4.2 mIU/l) and free T4 (fT4) and those with TSH above ULN were excluded from our study. Note that women with TSH above ULN had a further measurement of thyroperoxidase and thyroglobulin antibodies. Likewise, TRH dynamic test was not performed in any patient of our cohort.

The study was approved by the data protection department of Aix-Marseille University (PADS authorization number 2020-136**)**.

### Imaging Procedures

Pelvic ultrasounds were performed by skilled radiologists, either by suprapubic or transvaginal way, and determined the number of follicles, the ovarian volume as well as the ovarian surface. The latter likely have a slightly higher diagnostic performance than ovarian volume, when using a threshold of 5.5cm2 (12). The threshold of ≥ 20 follicles in, at least, one ovary was used as a criteria of PCOS. When performed, pituitary MRI was systematically done before pituitary dynamic tests (LHRH and metoclopramide tests), and read by an expert radiologist.

### Hormonal Assessment

#### Standard Hormonal Evaluation

The PRL measurement performed in the endocrine laboratory of the University hospital of Marseille used the Roche automaton COBAS 6000 e-601, which is a sandwich electrochemiluminescence immunoassay. This assay was approved by the COFRAC (French Comity of Certification) according to the norm ISO EN NF 15189 and has the following accuracy: 0.012% at 14.617 ng/ml and 1.28% at 34.169 ng/ml. For every women with a PRL value above ULN in our laboratory, another measurement was performed, or we asked for a previous value, that could have been performed in another laboratory.

For steroid hormones (estradiol, testosterone, DHEAS and 17-OH-progesterone), results are normalized as compared to ULN (because of normal ranges which differ, depending on the period of the patient’s cycle or age).

Every women with a 17-OH-Progesterone ≥ ULN underwent an ACTH Stimulation test (Synacthene^®^ 0.25 mg). If the latter confirmed possible congenital non classic adrenal hyperplasia, the patient was excluded from the analysis.

#### Dynamic Hormonal Testing

##### Metoclopramide Test

Metoclopramide test was performed in the outpatient facility of the endocrine department (daily hospital), to help distinguishing adenomatous from non-adenomatous causes of hPRL, as previously described (12, 13). Practically, after patient’s consent, the test started by a measurement of PRL before (T0) iv administration of 10 mg of metoclopramide, followed by a measurement at 30 (T30) and 60 minutes (T60) post-injection, respectively. For the interpretation, we calculated the relative increase of PRL, as follows: (PRL peak value - PRL baseline value) x100/PRL baseline value. The response was considered negative if the increase was < 100%, weak if the increase remained < 300% and positive if the increase was ≥ 300%. In case of PRLoma, the response is usually negative or weak, whereas it is positive in other causes of hPRL (14).

##### LHRH Test

All LHRH tests were performed in our daily hospital unit, after patient’s consent. A baseline measurement (T0) of LH and FSH was performed, followed by an injection of 100 μg of GnRH (Gonadorelin^®^) and subsequent measures of at 30 (T30) and 60 minutes (T60).

We calculated the LH/FSH ratio which should be below 1, but is usually reported as being ≥ 1 in PCOS women (14). In addition, we also used the LH T30/LH T0 ratio to assess the intensity of the LH stimulation. This ratio is likely ≥ 5 in women with PCOS (14).

### Statistics

Descriptive statistics, including the frequency and percentage for categorical variables, and the mean and standard deviation for continuous variables, were used to describe the cohort of women with PCOS.

Sociodemographic and clinical characteristics were compared between the cohort of PCOS/hPRL women and the cohort of PCOS/nPRL women, using Student’s t-test or Mann-Whitney test for continuous variables, and Chi-squared test or Fisher’s exact test for categorical variables when appropriate. A ROC curve was established to compare PRL threshold of interest for the etiological diagnostic of hPRL.

Spearman’s rank correlation coefficient between AMH and prolactin was estimated. All tests were two-sided. A p value ≤ 0.05 was considered statistically significative. All statistical analyses were performed using the software SPSS, version 20.0 (SPSS, Inc., Chicago, Illinois).

## Results

### Characteristics of PCOS Women

**Figure 2:**
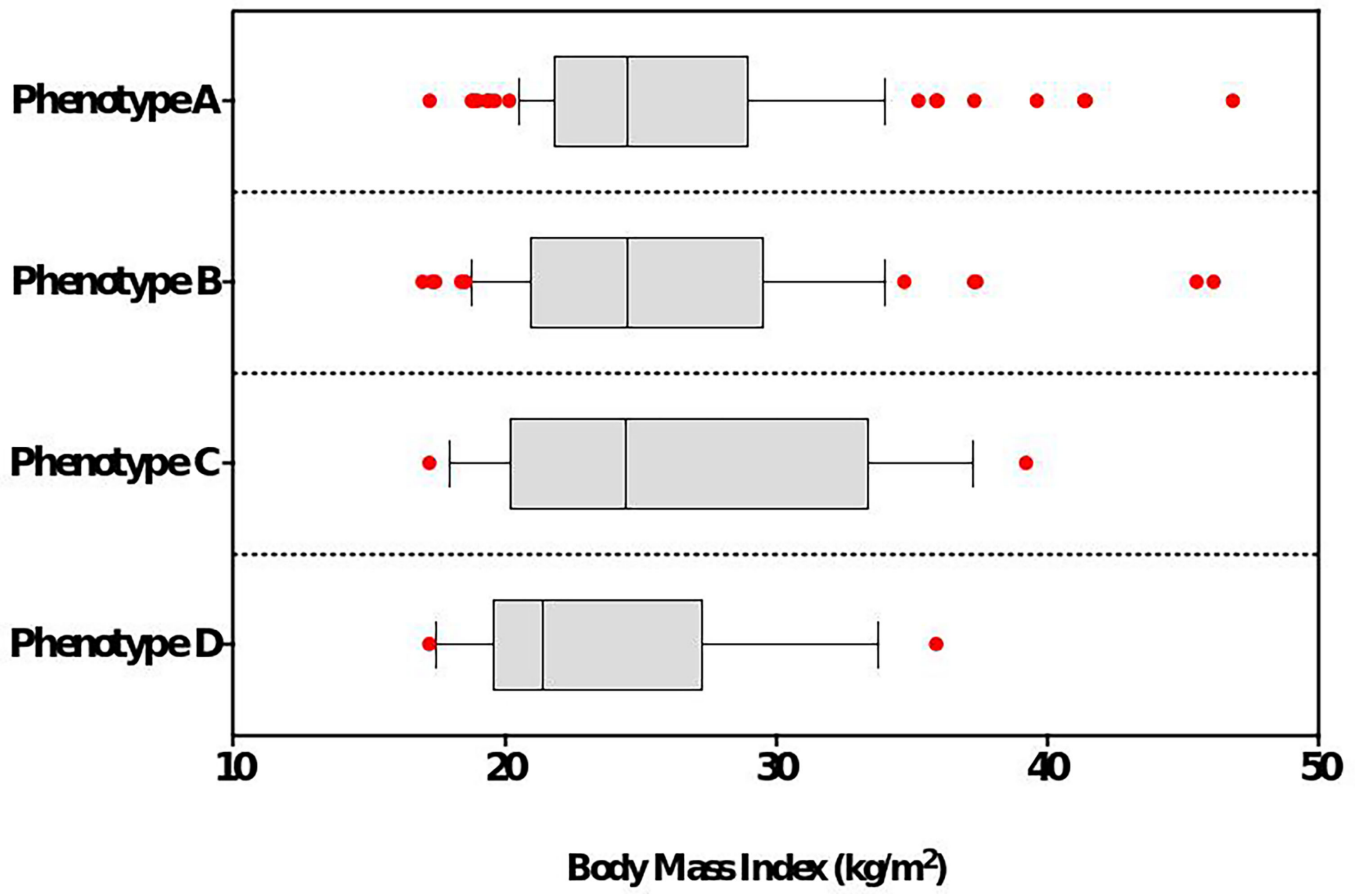
BMI (kg/m^2^) according to PCOS phenotype.

A total of 430 women with clinical symptoms suggestive of PCOS (oligo-anovulation, hyperandrogenism, menstrual irregularities, hirsutism, acne) were selected in our computer database (**Figure 1**), among whom 179 met the 2018 diagnosis criteria of PCOS. The mean age at inclusion was 23.8 ± 5.2 years and the mean body mass index (BMI) was 25.8 ± 6.1 kg/m^2^. Based on their BMI, they were classified as underweight (n = 10, 5.6%), of normal weight (n = 85, 47.5%), overweight (n = 43, 24%) or obese (n = 41, 22.9%). Regarding PCOS phenotypes, 48% had a phenotype A, 33% phenotype B, 10.1% phenotype C, and 8.9% a phenotype D (**Table 2**). We did not observe a significant association between the PCOS phenotype and BMI of the patient (**Figure 2**, p = 0.35). In the whole cohort, 161/179 (89.9%) women had menstrual irregularities, 163/179 (91%) had either a clinical and/or biological hyperandrogenism and 120/179 (67%) had ultrasonographic criteria for PCOS. The characteristics of the whole PCOS population of patients are summarized in **Table 2**.

**Table 2:** Description of the total cohort of the study.

Characteristics	Total cohort (n = 179)
**Age, y**	
Mean ± SD	23.8 ± 5.2
Median (range)	22 (18 – 40)
**BMI, kg/m^2^**	
Mean ± SD	25.9 ± 6.1
Median (range)	24.4 (17.0 – 46.9)
BMI classes, n (%)	
Underweight	10 (5.6)
Normal	85 (47.5)
Overweight	43 (24)
Obese	41 (22.9)
**PCOS phenotype, n (%)**	
A	86 (48)
B	59 (33)
C	18 (10.1)
D	16 (8.9)
**Menstrual disorders, n (%)**	161 (89.9)
Spaniomenorrhea	94 (52.5)
Amenorrhea	67 (37.4)
Missing data	18 (10.1)
**Hyperandrogenism, n (%)**	163 (91.1)
Clinical	142 (79.3)
Biological	106 (59.2)
**PCOM, n (%)**	120 (67)
AFC	
Mean ± SD	17.3 ± 9.2
Median (range)	15.5 (3.5 – 67.5)
Ovarian Surface, cm2	
Mean ± SD	5.5 ± 1.8
Median (range)	5.2 (2.0 – 10.6)
**Total testosterone, ULN**	
Mean ± SD	1.01 ± 0.52
Median (range)	0.87 (0.10 – 2.86)
**17-OH-Progesterone, ULN**	
Mean ± SD	0.57 ± 0.27
Median (range)	0.53 (0.14 – 1.6)
**Delta-4-Androstenedione, ng/ml**	
Mean ± SD	2.44 ± 1.14
Median (range)	2.24 (0.32 – 7.50)
**DHEA-S, ULN**	
Mean ± SD	0.83 ± 0.45
Median (range)	0.80 (0.06 – 2.94)
**Estradiol, ULN**	
Mean ± SD	0.56 ± 0.52
Median (range)	0.45 (0.05 – 3.13)
**AMH, ng/ml**	
Mean ± SD	6.63 ± 4.29
Median (range)	5.66 (0.67 – 22)
**Prolactin, ng/ml**	
Mean ± SD	18.80 ± 16.78
Median (range)	14.80 (4.90 – 153.3)
**LH/FSH**	
Mean ± SD	1.80 ± 1.20
Median (range)	1.58 (0.03 – 7.21)

AMH, Antimullerian Hormone; BMI, Body Mass Index; PCOM, Polycystic Ovarian Morphology; AFC, Antral Follicle Count; DHEA-S, Dehydroepiandrosterone sulfate; ULN, Upper Limit of Normal.

### Prolactin and PCOS

A total of 50 patients (28%) had at least one value suggesting hPRL, among whom 21 (42%) had a second measure confirming hPRL. According to our definition, the overall prevalence of hPRL in our PCOS cohort was 11.7%.

**Figure 3:**
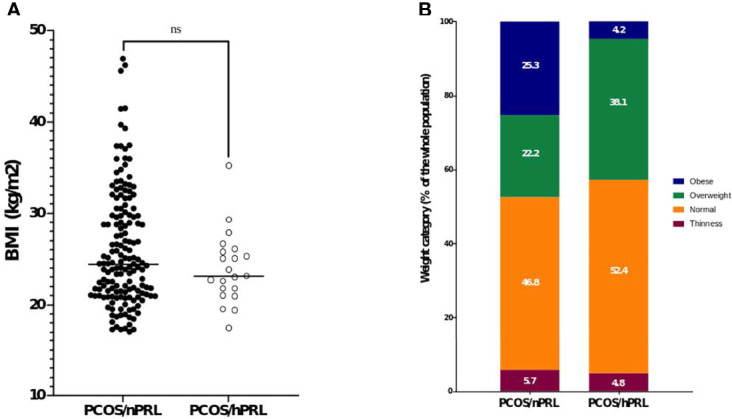
Characteristics of weight in the two cohort of PCOS women. **(A)** mean BMI according to the presence of hPRL or not and **(B)** weight category according to the presence of hPRL or not. ns, non significant.

Clinical and biological features of the two populations of patients (PCOS/nPRL vs. PCOS/hPRL) are described in **Table 3**. Provoked galactorrhea was found in 4 PCOS/nPRL patients and not in the PCOS/hPRL group (p = 0.54). Obesity was observed in 1/21 (4.8%) PCOS/hPRL patients as compared to 40/158 (25.3%) in the PCOS/nPRL group, while mean BMI did not differ significantly between the two populations of patients (**Figures 3A, B** p=0.297). Amongst the 21 patients with hPRL, PCOS phenotype A, B and C were found in 9 (42.8%), 9, (42.8%) and 3 patients (14.3%), respectively. There was no phenotype D.

**Table 3:** Univariate analysis between PCOS/nPRL cohort and PCOS/hPRL cohort.

Characteristics	PCOS/nPRL (n = 158)	PCOS/hPRL (n = 21)	*P* value
**Age, y**			*P*=0.977
Mean ± SD	23.8 ± 5.23	23.9 ± 5.44	
Median (range)	22 (18 – 40)	23 (18 – 35)	
**BMI, kg/m^2^**			*P*=0.297
Mean ± SD	26.10 ± 6.33	23.98 ± 3.91	
Median (range)	24.46 (17.00 – 46.88)	23.15 (17.47 – 35.25)	
**PCOS phenotype, n (%)**			NS
A	77 (48.7)	9 (42.9)	
B	50 (31.6)	9 (42.9)	
C	15 (9.5)	3 (14.3)	
D	16 (10.1)	0 (0)	
**Menstrual disorders, n (%)**	143 (90.5)	18 (85.7)	NS
Spaniomenorrhea	84 (58.7)	10 (55.6)	
Amenorrhea	59 (41.3)	8 (44.4)	
**Hyperandrogeny, n (%)**	142 (89.9)	21 (100)	NS
Clinical	122 (85.9)	20 (95.2)	
Biological	92 (65.7)	14 (66.7)	
**PCOM, n (%)**	108 (76.6)	12 (70,6)	*P*=0.559
AFC			*P*=0.292
Mean ± SD	17.61 ± 9.23	15.19 ± 9.10	
Median (range)	15.5 (3.5 – 67.5)	13.5 (5 – 40)	
Ovarian Surface, cm2			*P*=0.634
Mean ± SD	5.50 ± 1.86	5.04 ± 1.66	
Median (range)	5.35 (2 – 10.61)	5.12 (2.16 – 7.63)	
**Total testosterone, ULN**			*P*=0.268
Mean ± SD	1.03 ± 0.54	0.85 ± 0.37	
Median (range)	0.88 (0.10 – 2.86)	0.76 (0.3 – 1.77)	
**17-OH-Progesterone, ULN**			*P*=0.250
Mean ± SD	0.56 ± 0.27	0.60 ± 0.21	
Median (range)	0.51 (0.14 – 1.60)	0.55 (0.28 – 1.14)	
**Delta-4-Androstenedione, ng/ml**			*P*=0.144
Mean ± SD	2.40 ± 1.13	2.72 ± 1.21	
Median (range)	2.20 (0.32 – 7.50)	2.80 (0.32 – 4.80)	
**SDHEA, ULN**			*P*=0.243
Mean ± SD	0.82 ± 0.46	0.90 ± 0.28	
Median (range)	0.79 (0.06 – 2.94)	0.84 (0.47 – 1.40)	
**Estradiol, ULN**			*P*=0.852
Mean ± SD	0.54 ± 0.49	0.70 ± 0.78	
Median (range)	0.46 (0.05 – 3.13)	0.41 (0.06 – 2.99)	
**TSH, mUI/L**			*P*=0.826
Mean ± SD	2.26 ± 1.31	2.25 ± 1.22	
Median (range)	1.95 (0.41 – 8.39)	2.10 (0.54 – 5.79)	
**AMH, ng/ml**			*P*=0.190
Mean ± SD	6.76 ± 4.29	5.55 ± 4,.27	
Median (range)	5.80 (0.93 – 22)	5.12 (0.67 – 15.30)	
**Prolactin, ng/ml**			***P* < 0.001**
Mean ± SD	14.25 ± 4.96	53.03 ± 30.28	
Median (range)	14.05 (4.9 – 23.9)	46.2 (24.7 – 153.3)	
**LH/FSH T0**			*P*=0.655
Mean ± SD	13.31 ± 63.71	6.41 ± 4.56	
Median (range)	4.42 (1.88 – 663)	4.49 (2.15 – 16.45)	

AMH, Antimullerian Hormone; BMI, Body Mass Index; PCOM, Polycystic Ovarian Morphology; AFC, Antral Follicle Count; DHEA-S, Dehydroepiandrosterone sulfate; ULN, Upper Limit of Normal.

Bold value means comparison reached significance. NS, Non significant.

At the biological level, PCOS/hPRL women had a lower mean AMH level (5.5 ± 4.2 ng/ml) as compared to women from the PCOS/nPRL cohort (6.76 ± 4.1 ng/ml), even though, this difference did not reach significance (p=0.190). We did not observe any correlation between the levels of AMH and PRL, neither in the whole cohort (r = -0.05, p = 0.5), nor in the PCOS/hPRL group (r = 0.06, p = 0.8). We did not observe statistically significant differences in the ultrasonographic ovary characteristics of hPRL/PCOS as compared to nPRL/PCOS women (**Table 3**), and, moreover, we did not find specific ultrasonographic features related to hyperprolactinemia.

Finally, women of the PCOS/hPRL group had a trend for a less explosive response to the LHRH test (mean LH T30/LH T0 ratio = 6.4) as compared to women of the PCOS/nPRL group (mean LH T30/LH T0 ratio =13.31) however without reaching statistical significance (p=0.671).

### Etiologies of hPRL

**Figure 4:**
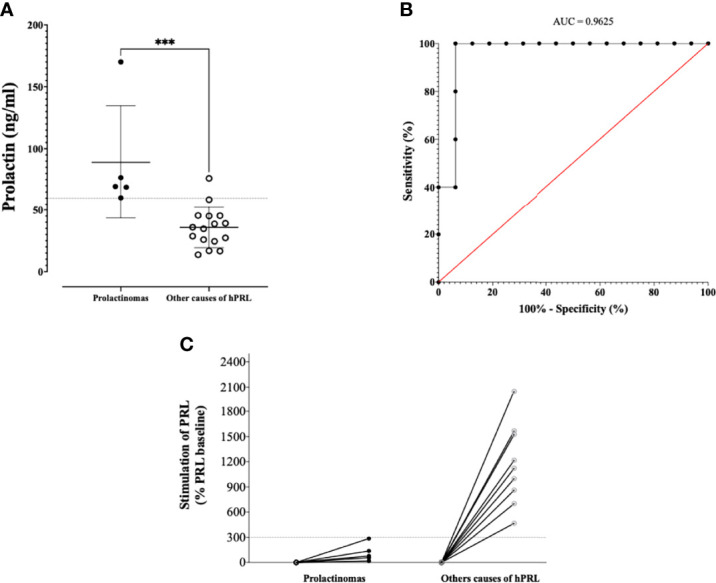
**(A)** PRL levels according to the presence of a PRLoma or another hPRL cause. The dashed line indicates a threshold of PRL of 60 ng/ml. **(B)** ROC curve assessing the statistic performances of basal PRL to distinguish PRLoma from others causes of hPRL **(C)** Metoclopramide test results, with relative increase according to the presence of a PRLoma or another hPRL cause. ***p < 0.001.

Five out of 21 (23.8%) women of the PCOS/hPRL group had a microprolactinoma. The mean diameter of the pituitary lesion was 4.4 ± 1.7 mm and the mean PRL value in this subgroup of women was 85 ± 45 ng/ml, significantly higher than the mean value in women with other causes of hPRL (35.7 ± 16.2 ng/ml, p < 0.001, **Figure 4A**). Based on the ROC curve, sensitivity/specifity of a PRL threshold of ≥ 60 ng/ml for the diagnosis of microprolactinoma as compared to other causes of hyperprolactinemia in PCOS women, was 100% (95% CI:56.5% -100%) and 93.7% (95% CI:71.7- 99.7%), respectively (**Figure 4B**, AUC = 0.962). All of these women were, subsequently, treated with dopamine agonist therapy which normalized PRL and decreased the size of the pituitary lesion. We did not identify patients with a macroprolactinoma or pituitary tumors responsible for a disconnection hPRL. Five patients (23.8%) had a macroprolactinemia, and one of them underwent a pituitary MRI before macroprolactinemia was ruled out. The MRI showed a 2.4 mm lesion which, after reexamining images, was uncertain. Four patients (19%) had an iatrogenic hPRL (the use of medications was confirmed after a phone call). One patient (4.8%) had hPRL likely due to hypothyroidism after reexamination of her medical record, as her TSH reached 5.8 mUI/l on one measurement. However the link between hPRL and hypothyroidism is more obvious for TSH ≥ 7.5 mIU/l (15). Finally, 6 (28.6%) had an idiopathic hPRL, despite complete investigations (PRL chromatography, pituitary MRI, hypothyroidism, pregnancy, hPRL drugs) and a careful analysis of their medical files. In this subgroup, the mean PRL value was 33.4 ± 9.3 ng/ml.

A metoclopramide test was available in 14 patients with hPRL, including the 5 microprolactinomas. The test resulted in a stimulation of baseline PRL of less than 300% in all PRLomas, while it was above this cut-off in all the other etiologies (**Figure 4C**). In particular, the patient with the 2.4 mm pituitary lesion and macroprolactinemia, had a stimulation of her baseline PRL of 1218%.

**Figure 5:**
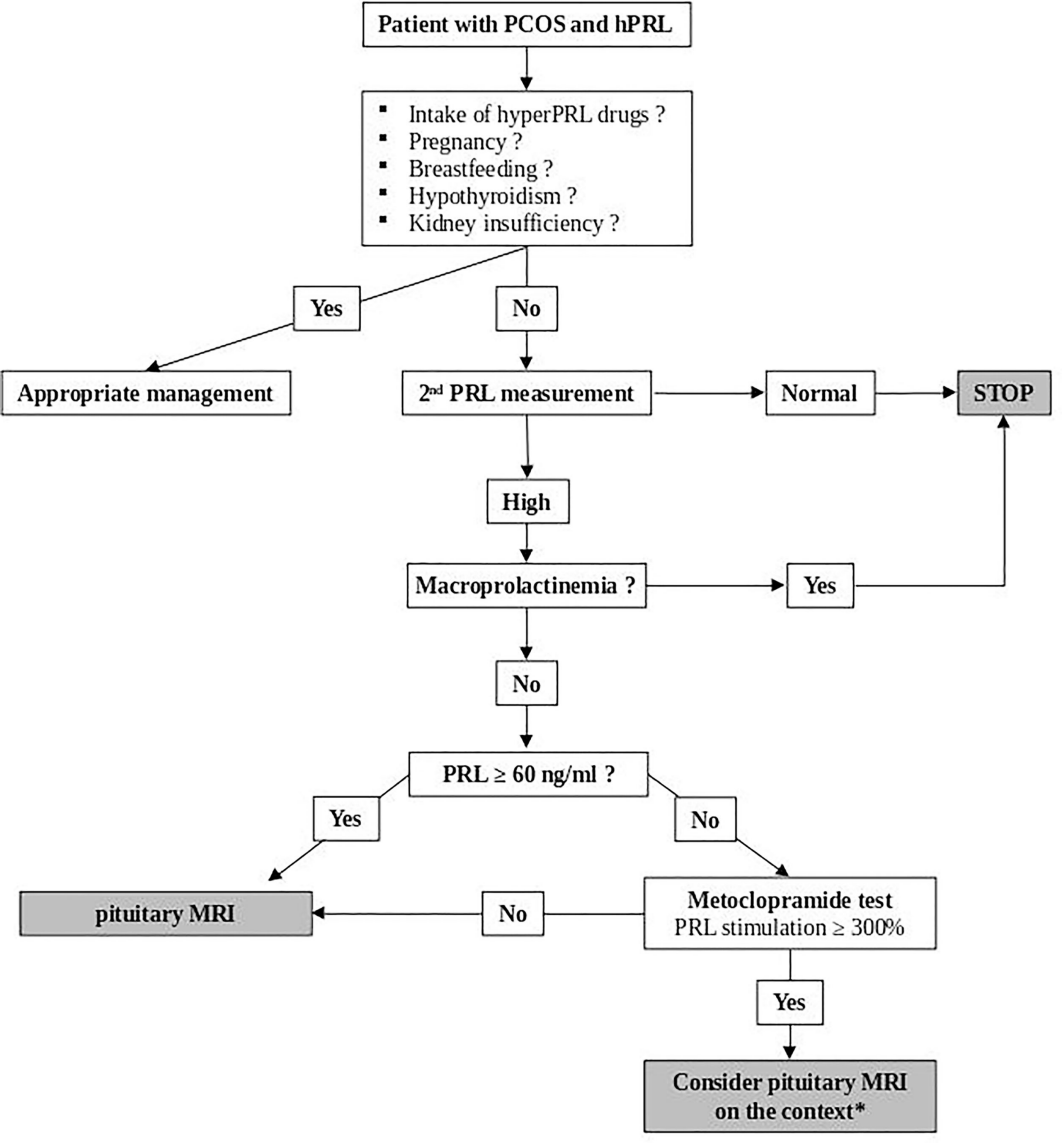
Decisional algorithm for the etiology of hPRL in PCOS women. *Pituitary MRI is advised in case of pituitary tumoral syndrome, dopamine agonist intention-to-treat or abnormalities of other pituitary hormones.

In summary, our results suggest that performing metoclopramide test in PCOS women with hPRL, especially in those with value that remain below 60 ng/ml, could be a helpful tool for discussing the relevance of a pituitary MRI (**Figure 5**).

## Discussion

The association of hyperprolactinemia (hPRL) and polycystic ovarian syndrome (PCOS) is not uncommon and raises essential questions for the therapeutic management of the patient. One can easily assume that information delivered to the patient, therapeutic outcome and follow-up strategy will substantially differ if a prolactin-secreting pituitary adenoma is diagnosed in addition to PCOS. In our study, confirmed hPRL was observed in 11.7% of PCOS women, however the reason for this hPRL remained unsolved for a quarter of them. Whether a pathophysiological link exists between PCOS and hPRL has been and is still widely discussed in the scientific community (16–18). The rapid evolution of knowledge about both PCOS and hPRL renders studies conducted before the 2000s, obsolete. Most of these studies were published before consensual diagnosis criteria of PCOS, while others used only one blood sample showing hPRL with no control, and finally, in another subset of studies, screening for macroprolactinemia was inconsistent. There are several mechanisms that have been proposed to explain the increase of PRL in PCOS. One of them is the alteration of the dopamine turnover, responsible for an increase in PRL secretion, a deteriorated GnRH output and, eventually, an abnormal ovary function (19). Likewise, high levels of estrogens, similar to those found in PCOS, are known to stimulate the synthesis and secretion of PRL, as well as proliferation of lactotroph pituitary cells in animals (20–22). However, this relationship in humans is controversial (23–25) and, our study does not support this hypothesis since estrogen levels did not differ significantly between PCOS women with and without hPRL. High PRL levels have been associated with alterations in steroidogenic enzyme activities, increased androgen production, insulin resistance, hirsutism and PCOS morphology (26, 27), all of the above being essential components of the PCOS phenotype.

None of the hormonal factors we collected significantly differed between PCOS/nPRL and PCOS/hPRL women. However, we hypothesized that hPRL patients could have a less pronounced phenotype of PCOS, as PRL is known to exert a negative feedback action on the pulsatility of GnRH and the release of LH and FSH. We did not observe clinical or biochemical differences between the two populations of patients in our study with the exception of a higher proportion of obesity in the PCOS/nPRL group as compared to patients with hPRL. Should this be interpreted as a less pronounced metabolic syndrome phenotype is undetermined yet, however the metabolic aspect of PCOS continues to question experts of the field and leads to innovative pathophysiological links and statements, like the one recently published by Aversa et al. (28). Similarly, while not significant, we observed both a trend for a less explosive response to the LHRH test in hPRL patients as compared to those with nPRL, and a lower AMH. Again, the negative feedback exerted by PRL at the pituitary level is a plausible explanation of this biological profile.

Another aspect of our study that must be emphasized is that more than a half of the patients with one single high value of PRL eventually had a normal value on a second sample. Because the context of the patient (i.e., oligo-anovulation, menstrual irregularities, desire for pregnancy) prompts clinicians to screen for an organic disease, it remains essential, in a first-step diagnosis procedure, to confirm hPRL on a second sample before any further investigations. The prevalence of prolactin-secreting pituitary adenoma (PRLoma) in our whole cohort was found to be 2.8%, slightly higher than the one reported in the literature (0.3 - 0.5%) (7). This difference makes sense, since all of our patients were selected with a systematic measure of prolactin (unlike in general population), and because the real prevalence of microprolactinoma in young women is likely to be underestimated (29). Our patients with PRLomas had a significantly higher value of PRL and all of them had a PRL value ≥ 60 ng/ml. A previous study suggested that a PRL threshold of 85.2 ng/ml could distinguish patients with PRLoma with 77% sensitivity and 100% specificity (30). Using this threshold in our study would have conducted to misdiagnose PRLoma in 4 out of 5 patients, underlying the fact that no optimal cutoff exists for the distinction of PRLomas and other causes of hPRL, and/or that cut-off values may differ depending on the characteristics of the assay used. In this setting, the use of dynamic tests, like metoclopramide injection, could be helpful. The metoclopramide test has been developed in the 70’s as a strong stimulator of PRL secretion (31) and is used to distinguish hPRL due to pituitary adenoma from other causes of hPRL (32), with a cut off of 300% being admitted as a limit of PRL stimulation beyond which hPRL is unlikely due to PRLoma (14). A previous study showed that the metoclopramide-induced stimulation of PRL secretion was preserved in PCOS women, with a ratio of stimulated/basal PRL up to 1000% (33). However, no previous work investigated the added value of the metoclopramide test in PCOS women with hPRL. As such, our study is the first to show that the metoclopramide test is helpful to distinguish hPRL secondary to microprolactinoma as compared to other causes of hPRL in PCOS women, and therefore could be an interesting tool for discussing the relevance of a pituitary MRI.

Our study has obvious limitations due to its retrospective nature. The monocentric enrollment of patients is naturally subjected to a bias of inclusion, even more because we included patients from endocrinology and medically assisted procreation departments. A high prevalence of PRLoma is, therefore, not that surprising since most patients complained of menstrual irregularities and/or oligo-anovulation, a bias that could slightly overestimate the real prevalence of PRLoma in PCOS women. Investigating the PRL level of PCOS women visiting a dermatologist for acne and/or hyperseborrhea, without menstrual disturbances, could be of interest in an upcoming study. The retrospective design of our study is another limitation as it can be a cause of missing data. Nonetheless, we also would like to highlight the strengths of our work. First, we accumulated data from many well-characterized women meeting the Rotterdam criteria to establish our reference population. Biological data were obtained through the intervention of the same paramedical team, treated in the same biological laboratory, interpreted by the same clinicians. Accordingly, we meticulously analyzed every medical file to enrol women that had a clear diagnosis of PCOS. We thus selected 179 out of 430 patients, and thereafter proceeded with the same methodology for hPRL patients. Those patients had at least 2 values of PRL above the limit of normal to be considered as being hyperprolactinemic. This arbitrary decision, which resulted from our aim to focus on “real” hPRL patients, allowed us to select 21 patients, and, at the same time, turned away 29 patients from this definition.

Overall, the prevalence of macroprolactinemia in our PCOS/hPRL population reached 24%, roughly similar to what is reported in the literature (34) confirming the fact that macroprolactinemia must be ruled out in priority, even in PCOS women, before proceeding with further investigations. A non-negligible subset of patients (28% in the PCOS/hPRL cohort) had an idiopathic hPRL based on our diagnostic criteria, and despite extensive investigations. Pituitary MRI was normal and mainly performed to rule out the possibility of a disconnection hPRL. These idiopathic cases had mild elevations of PRL and, importantly, all of them had a marked response (i.e., > 300% from baseline PRL) to the metoclopramide test, thus not in favor of picoprolactinomas (i.e., maximal size of adenoma < 3 mm, usually not visualized on pituitary MRI). Whether a subtle interaction between PCOS pathophysiology and the mechanisms of PRL secretion occurs in these cases still needs to be deciphered.

In conclusion, our study shows that hyperprolactinemia is often present in lean PCOS women, occurring in almost 12% of cases. This suggests that a dedicated decisional algorithm to manage these patients may be helpful to treat patients with microprolactinomas and avoid unnecessary and costly diagnostic procedures in others (**Figure 5**).

## Data Availability Statement

The raw data supporting the conclusions of this article will be made available by the authors, without undue reservation.

## Ethics Statement

Ethical review and approval was not required for the study on human participants in accordance with the local legislation and institutional requirements. Written informed consent for participation was not required for this study in accordance with the national legislation and the institutional requirements.

## Author Contributions

Conceptualization, CR and TC. Validation, BC, TB, and TC. Writing—original draft preparation, CR and TC. Writing—review and editing, CR, BC, MV, BF, NR, TB, and TC. Statistics, SF and NR. Supervision, TB and TC. All authors have read and agreed to the published version of the manuscript.

## Conflict of Interest

The authors declare that the research was conducted in the absence of any commercial or financial relationships that could be construed as a potential conflict of interest.

## Publisher’s Note

All claims expressed in this article are solely those of the authors and do not necessarily represent those of their affiliated organizations, or those of the publisher, the editors and the reviewers. Any product that may be evaluated in this article, or claim that may be made by its manufacturer, is not guaranteed or endorsed by the publisher.
